# Classification and Production of Polymeric Foams among the Systems for Wound Treatment

**DOI:** 10.3390/polym13101608

**Published:** 2021-05-16

**Authors:** Paolo Trucillo, Ernesto Di Maio

**Affiliations:** 1 Department of Chemical, Material and Industrial Production Engineering (DICMAPI), University of Naples Federico II, Piazzale Vincenzo Tecchio 80, 80125 Napoli, Italy; edimaio@unina.it; 2 IODO S.r.l., 84123 Salerno, Italy

**Keywords:** wound treatment, patches, foams, foaming process, supercritical fluids

## Abstract

This work represents an overview on types of wounds according to their definition, classification and dressing treatments. Natural and synthetic polymeric wound dressings types have been analyzed, providing a historical overview, from ancient to modern times. Currently, there is a wide choice of materials for the treatment of wounds, such as hydrocolloids, polyurethane and alginate patches, wafers, hydrogels and semi-permeable film dressings. These systems are often loaded with drugs such as antibiotics for the simultaneous delivery of drugs to prevent or cure infections caused by the exposition of blood vessel to open air. Among the presented techniques, a focus on foams has been provided, describing the most diffused branded products and their chemical, physical, biological and mechanical properties. Conventional and high-pressure methods for the production of foams for wound dressing are also analyzed in this work, with a proposed comparison in terms of process steps, efficiency and removal of solvent residue. Case studies, in vivo tests and models have been reported to identify the real applications of the produced foams.

## 1. Introduction

**Figure 1:**
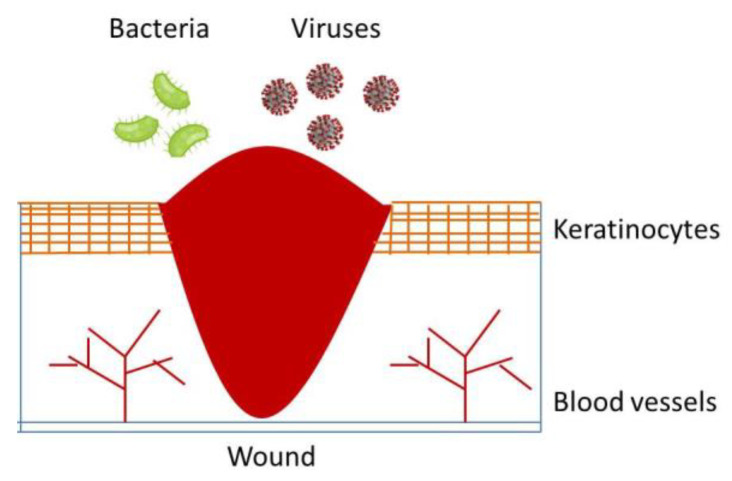
A sketch of a general wound.

Wounds could be defined as a skin discontinuity or break due to a mechanical, thermal or physical damage caused by an external event [1]. According to the literature and to the daily medical experience, wounds can be generated by the effects of accidental or inferred contact with blades [2], fire burns [3] and gunshot [4]. These events are passively suffered by the target organs; in other situations, they can be induced by surgical operations, under controlled area and conditions such as anesthesia [5]. The normal anatomic structure of skin is characterized by a continuous tissue that functions to protect human beings from external agents in order to preserve the integrity of the organs and tissues. According to this definition, a wound can be intended as a disruption of this continuous skin-tissue structure (see Figure 1, where a section of a wounded skin is qualitatively represented).

The healing process has encountered an evolution during the human ages. However, the main objective of wound dressing has always been, of course, to stop the loss of blood and avoid infections, as first aid to a wounded person. Indeed, the ancients knew that many traditional plants showed antibacterial activity and anti-radical effects [6], since plant extracts contain potentially harmful essences. For this reason, it was important for physicians to being able to recognize and distinguish the powerful from harmful herbal extracts.

Several natural occurring materials such as animal fats, extracts obtained from plants and leaves fibers, and honey, were utilized for the treatment of wounds [7]. With the diffusion of new materials and the increased knowledge, gauzes made of cotton, wood or lint was used with a double function: prevent the introduction of bacteria causing infections and keep the wound as dry as possible. Indeed, scientists understood that a moist environment was not effective for wound healing. Currently, we have the possibility to use synthetic and composite materials and bandages aimed at fast healing; the discovery and frequent use of antibiotics added a biochemical intervention to support the healing process made by bandages [8].

The scope of this review paper is to describe the techniques utilized for the treatment of wounds with polymeric foams, from ancient to modern times, with the aim of focusing on the production techniques and on in vitro tests performance. Artificial and natural methods will be compared across the traditional medicine, exploring the effects and the advantages of drug loading for controlled delivery of antibiotics or healing agents. Conventional and novel foam production systems will be compared, giving examples and describing specific case studies.

## 2. Classification of Wound Healing Processes

Approaches to wound healing could be classified according to the nature of the repairing process, that is in this case, active or passive. In the first case, the intervention of dressings, patches or antibiotics is necessary to help accelerating the healing process; in the second case, the healing process is induced and completed autonomously by the wounded organ [9].

The wounds that can be healed completely by the body are called acute and represent the simplest and least dangerous [10]. They are characterized by minimal formation of scars and total healing in no more than 8 weeks [11,12]. Acute wounds are caused by frictional impacts among the skin and hard surfaces. Acute wounds could be caused also by fire explosions or normal fires, but also acute oxidations such as exposition of the skin to chemical agents, such as strong acids like nitric acid, sulfuric acid, hydrochloric acid, or hydrogen peroxide. Burns can be caused also by radiation, high voltage or other thermal sources [13,14]. Of course, in these last cases, the exposition time to the thermal source can cause less or more serious damage to the skin [15]. Chronic wounds are the second category of wounds that heal in a very long time (according to the literature and to daily experience of medical doctors, 12 weeks is the minimum time to consider a wound as chronic) [16]. This can be caused by other illnesses, such as diabetes which delays the healing process when the average glucose levels in the blood are higher than 300 mg/dL for a long time [17,18,19,20]. Further, infections already present in the body cannot help the healing process. Indeed, the wound cannot be healed if it is characterized by a wet surrounding environment. In the case of patients affected by obesity, wound healing is even slower, since there is a reduced blood flux; decubitus ulcers or bedsores are classified as chronic wounds, as well as leg ulcers caused by ischemic illness [21,22,23].

The severity of wounds is also determined by the penetration under the external layer of the skin. A wound that interests only few layers (epidermis) is called superficial and is easily healed; instead, a wound that involves the dermal layers and blood vessels are partially thick. If the wound reaches the subcutaneous fat and even deeper tissues, they are called fully thick. In particular, the superficial wounds concern the integument tissues such as skin, hair and glands; the thick wounds are followed by tissue loss, tissue death and diffusion of bacterial infections to the local tissues.

Wounds can be classified also in terms of their appearance [24,25,26]. Necrotic wounds are often characterized by a black or olive-green color, that is a precise indication of a dead dry tissue [27]. Sometimes these wounds can separate spontaneously from healthy tissues, thanks to the constant cleaning work of macrophages, that eliminate dead cells, separating them from living cells. In other cases, for example after fire-burns, the dead tissues need to be removed using surgical operations, that are much more painful for the patients.

A wound can be sloughy, i.e., characterized by fluid and moist necrotic tissues, generally of yellow color. This state of wound is determined by inflammation causing exudations, and it is characterized by very long healing times, not always successful [28]. Granulating wounds are red or dark pink in color, and they are the most typical wound in the proliferative phase [29]. This is easily healed with proper medication and without incurring of infections. Then, epithelializing wounds are pink and generally regard the situation of a healing process that is successful and almost completed. It is worth saying that the so named infected wound is characterized by hot tissue, red color, with the production of pus exudates; these last are caused by the response to the presence of infections caused by bacteria proliferation in situ. This is, of course, the cause of a delay in wound healing process [30].

## 3. Biological Wound Healing Process

The human body is programmed as a living machine that has all the protocols aimed at self-adjusting and recovering the previous equilibrium conditions. In case of wound occurrence, the first two operations that the body is programmed to perform are hemostasis and coagulation [31,32]. The first one is aimed at the most important function of preventing further loss of blood, in order to avoid immediate exsanguination. The second step consists in avoiding external cells to invade too deeply the skin tissue and blood vessels. This can be performed through the creation of a natural matrix to join the internal layers of the skin that works as a barrier for bacteria and viruses [33,34,35].

Hemostasis is guaranteed by the production of fibrin in the local site of the wound, and the deposited amount is determined by a very strict balance among coagulation process and vasoconstriction. The last one is characterized by a restriction of the blood vessel to reduce the blood flow rate locally. Vasoconstriction is a neural reflex, that occurs immediately as soon as the brain understands through the pain that a wound has been generated. The fibrin creates this matrix, that helps preventing introduction of external elements and supports homeostasis mechanism, aimed at the creation of a clot [36,37,38].

## 4. Local and System Factors Affecting Wound Healing

The process of wound healing is activated by natural mechanisms; however, there are several local and systemic factors that can affect the natural process of healing [39]. Concerning local effects, the most two important are oxygenation and infection [40,41,42]. Currently, it is very well known that the role of oxygen is extremely important for cellular metabolism and the consequent production of energy to let the body perform all the operations that characterize the daily life [43].

Among these operations, it is absolutely important to include wound healing; energy is highly requested for supporting this process. In case of a reduction of energy production, the wound healing process could become slower or in worst cases stopped, with very dangerous consequences for the patients. In the wounded sites, the disruption of skin tissues and blood vessels causes the depletion of oxygen, resulting in local hypoxia. Indeed, in the wounded places, the oxygenation is not continuously restored as in the other tissues. Hypoxia activates macrophages to prevent infections, generating fibroblasts and producing cytokines to induce cell proliferation. However, if the oxygen levels are quickly restored, the wound healing process could be endangered. Indeed, microorganisms could contaminate the wound and adjacent healthy tissues, colonizing them and causing inflammation.

Then, there are systemic factors that could significantly affect the process of wound healing. One of these is age; old people do not have a fast response of wound healing; moreover, an advanced age can alter the anti-inflammatory response and collagen synthesis compared to young aged system responses. Another important factor affecting wound treatment is characterized by diabetic disease. Diabetes is a life-long chronic disease that deteriorates the organs slowly. In the case of the poor control of diabetes, the high level of glucose in the blood can delay and impair the normal wound healing process. In particular, an even worse control of diabetes results in foot ulcers [44] that in most cases results in amputation or in a total reduction of the quality of life [45]. High levels of glucose, also known as hyperglycemia, cause oxidative stress in diabetic patients, increasing the wound recovering period up to 60 times more than a non-diabetic patient [46,47].

However, there are other external agents that affect wound healing process, such as the consumption of alcohols and smoking of cigarettes [48,49]. Alcohol causes interferences with the mechanisms of defense and makes the human body more vulnerable to infections caused by wounds. Concerning smoking, nicotine causes a reduction of blood perfusion, increasing the carbon monoxide concentration and compromising the strict balance with oxygen consumption.

## 5. History of Wound Treatment

The first medical testimonials are reported on papyri found in the ancient Egypt [50]. Ancient Egyptians well knew that wounds could be infected and the use of certain plants had antimicrobial effects. As examples, they knew that *Hypericum perforatum* could be used on perforating wounds, and *Symphytum officinale* had antibacterial and healing effects on wounds and fractures. Leaves were often used as bandages, sometimes accompanied by ointments; primitives knew that once washed, wounds could be self-dressed [51].

Civilizations such as the Greeks developed their medical knowledge gathering information from populations living in Mesopotamia or from famous Chinese medicine history, since the most legendary emperors were said to have a particularly high longevity. In Mesopotamia, wounds were treated with milk/water solutions and honey, with bandages of wool or linen [52]. However, the way of stopping the bleeding was not known yet.

Greek medicine took a lot from ancient populations, and also tried to develop the surgery, quite advanced for those ages. Therefore, during the narration of the Trojan War, the Greek army were said to have its own surgeon. People from Greece knew how to recognize necrotic tissues and how to remove them or make cauterizations or amputations [53].

The modern way of treating wounds dates back to Hippocrates, that is considered the father of the scientific approach to medicine. He was the first to propose to treat the contused wounds with salves to promote suppuration, eliminating necrotic tissues and decreasing the effects of inflammation. He recommended to wash the wound using wine or vinegar to eliminate bacteria, and then to keep the wounded tissue dry [54].

The use of bandages became really diffused around 500 B.C.; the surgeons knew that the bandage should be effective but not too tight, in order to avoid gangrene of the wound. Then, Romans developed their knowledge on the Greeks’ since they had no traditions in this art. In this period, the first sutures of fresh wounds after washing, and the treatment of infected wounds with the aim of converting them back to fresh tissues were finally considered. In particular, Galenus gave a great contribution using the polypharmacy approach for the treatment of wounds, making the Romans gain prestige for medicinal arts in the first two centuries after Christ [55].

In the middle ages there are books with entire chapters about surgery. Wounds were pressed with a moist sponge applied to them. Sometimes, the application of cobwebs was also proposed. If none of these were effective, the wound was cauterized. In case of the previous methods not succeeding, venous bleed was treated with styptics [56]. However, the first European school of medicine and surgery was settled in Salerno, in the South of Italy, in the ninth century [57,58,59]. For the first time, practitioners learnt from highly experienced physicians how to treat wounds and needed to be licensed before having the right to practice the medicinal arts.

The Italian medicine spread in France and Guy de Chauliac posed the modern approach to modern wound medical treatment [60,61], that consisted essentially in removing foreign bodies, re-approximate the separated parts of the tissue, perform maintenance on their apposition, conservation and treatment of the complications. Of course, a large discussion was raised when the gunshot power was introduced in Europe: cauterization came back as the most used technique. After Renaissance, surgery developed in its most modern concept and actuation.

**Figure 2:**
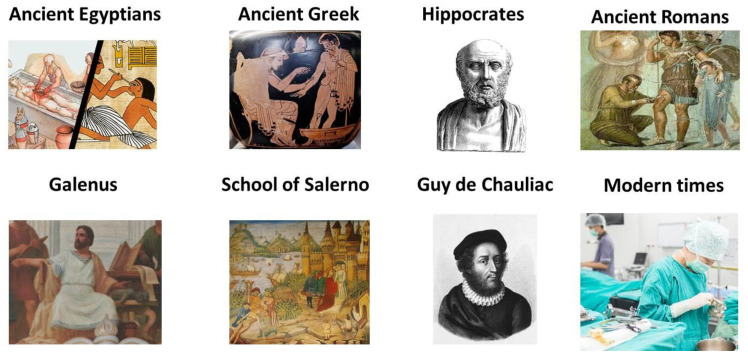
Main physicians and milestones of medicine for wound treatment from ancient to modern times (sources: www.wikipedia.com, www.youtube.com, accessed on 10 April 2021).

A brief history by icons evolution has been proposed in Figure 2.

## 6. Types of Wound Treatment in Modern Times

In recent years, wound dressing has been significantly modernized through the use of synthetic polymers that can be classified as passive or interactive [62]. The first ones are not occlusive; it is just the use of a polymeric film to cover the wound and help restoring the normal function of that tissue. Interactive dressings are occlusive, meaning that they cover properly the wound with the specific function to create a barrier against bacteria and viruses, intended as a sort of interface between the wound and the external world [63].

Some modern studies assessed that moist wound dressing promoted a faster healing rate compared to dry wound dressing. However, it was found also that, concerning the proliferation rate of cells during wound healing, neutrophils and macrophages multiply slowly in a moist wound. However, endothelial and fibroblast cells proliferate fast in a moist condition. Moreover, the movement of epithelial cells across wounds is favored by wet environment and absence of scabs. The use of occlusive dressings avoids exposure to external environment [64,65,66,67,68].

The commercially available wound dressing types could be divided into four main typologies: foams [69], hydrogels [70], alginates [71] and hydrocolloids [72]. All of these are characterized by a polymeric nature. Generally, the polymeric foams are used to treat chronic wounds, wounds caused by burns and surgery wounds [73]. Polymeric hydrogels are employed for the treatment of ulcers, but also for the preparation of chemotherapy peels. Polymeric alginates are used for thicker wounds due to burns, high exudate wounds and surgical wounds [74,75]. Finally, polymeric hydrocolloids are used to treat particularly chronic ulcers and, again, for burns and not particularly thick burns wounds.

## 7. Overview of Synthetic Polymeric Dressing Methods

Generally, biopolymeric materials are more desired for the treatment of wet wound dressings. However, these systems are expected to provide a good water and gaseous exchange, a good hemostatic power with high mechanical strength, a rapid wound healing, the highest possible pain reduction and the elimination and/or prevention of bacterial infections. A wound treatment system should be nontoxic, non-allergenic, biodegradable and biocompatible, and adhere biologically to the wound, anchored to the sane skin around the wound. Of course, another property that is often difficult to obtain together with the previous one, is the reduced cost of production and nursery application [76].

According to the wound healing management types, the use of polymeric film is employed as occlusive dressing. Polymer films can trap exudates, keeping the wound moist. Polyurethane (PU) is used in many dressing systems [77], since it has a double positive function: it provides a good barrier while permits a good permeability to oxygen. PU is impermeable to bacteria and liquid but contributes the exchange of moisture vapor with fresh air. However, the exudates can accumulate among the wound and the film, obliging to replace these films often. Bacteria cannot penetrate this environment. Wounds treated with PU often reach the condition of the formation of scabs, with the formation of a granulated tissue on the wound, that is a collagen-rich and vascularized tissue.

Another film-like dressing method is represented by hydrocolloid dressings [78], that are obtained from colloidal suspensions or solutions, such as gelification agents. These materials are generally combined with adhesives or elastomers, such as pectine, cellulose derivates and gelatins. These films are particularly versatile for clinical uses (for example, to treat leg ulcers) since they can adhere easily to dry surfaces and moist wound environments. They are characterized by a self-response therapy, due to their ability to produce a gel that covers the wound, and there is no pain associated when removed from the repaired wound. They are also successfully employed for pediatric uses and for chronic wounds.

The third type of wound dressing is characterized by alginate dressings, that are generally made by calcium and sodium salts of alginic acid, generally available in foam or fibers form [79]. Additionally, in this case gels can be formed on the wound due to alginate high absorbency power. In particular, this mechanism is activated by ion exchange phenomena among the fibers of alginate and the exudates or blood, that results in the formation of the protective film/foam. This will guarantee optimal moisture and temperature conditions. This ability is particularly efficient for calcium ions, that contribute to form crosslinked gel, providing a slow degradation and being ideal materials for the use of scaffold in tissue engineering. Therefore, the necrotic tissues can be replaced by the use of scaffold, that are obtained from the intelligent use of smart synthetic polymers; by substituting the skin, these polymers can enhance the healing process of wounds, leaving behind them the connective tissue, that follows its natural progress to restore naturally the wound.

Hydrogel dressings are obtained from hydrogels, that are hydrophilic such as pol(vinyl pyrrolidine) and poly(metacrylates). Sometimes they are used in combination with alginates, since they can compose the properties of the gel with the elasticity of alginate for the creation of the films [80]. In particular, hydrogels contain a 70–90% amount of water and, for this reason, they cannot absorb huge amounts of exudates. This disadvantage makes them useful only to dress light wounds with poor production of exudates. The accumulation of fluids, indeed, can cause replication of bacteria and infections inside the wound. On the other side, hydrogel dressings are not reactive with human tissues and also not irritating.

A kind of system to dress wounds that has been used for long times is the semi-permeable film dressings/patches [81], since they were made of nylon derivatives plus polyethylene materials in order to facilitate the occlusion phenomenon. However, nylon had a limited capability to absorb wound exudates, leading to a higher bacteria proliferation and to the necessity of a continuous substitution. Moreover, they tend to wrinkle when removed from their packages.

Scaffold and foams are generally used to deliver drugs or other active molecules, such as antibiotics, growth factors or even genetic material [82]. DNA delivery using these systems can for example regenerate ulcers caused by diabetes. It was also studied the possibility to both load antibiotics and recombinant growth factors together with derived dermal cells, in order to restore more easily the dead tissues while simultaneously avoiding infections. However, in some cases there is the possibility of antibiotic-resistant bacteria causing wound infections; in these cases, the normal loaded antibiotic would be not effective, and light activated photosensitizers may be employed to inactivate microorganisms by the formation of reactive oxygen species. Alginate can be involved in the formation of foams for topical delivery of curcumin, whose release is activated by photosensitive external stimuli. These kinds of foams have sterile properties and provides efficient administration without side effects to patients such as pain suffering.

Other kinds of wound dressing synthetic methods are characterized by wafers [83]. They are obtained from gel-forming polymers using the lyophilization method, creating a solid matrix. Their water physical phase is similar to the one of foams, absorbing wound fluids and transforming them into a gel that will keep the environment moist at the equilibrium. The importance of these materials stands in the use of additivities, such as the polyhydroxyalkanoates (PHA), whose nanocomposites are employed not only for wound healing, but also in in many fields of applications, such as tissue engineering, active packaging and drug delivery [84].

**Figure 3:**
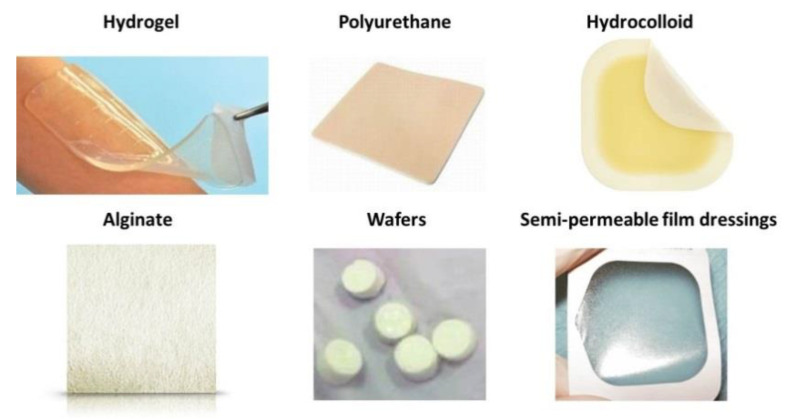
Types of wound treatment images.

The different wound treatment types described above were summarized by icons in Figure 3.

## 8. A Focus on Foams

Polymeric foams represent a unique technological platform to wound treatment, given their large versatility, that has brought them in the most diverse application fields. Foam versatility resides in the possibility to tune their structure, in terms of density and pore morphology, and formulation, in terms of the composition, shape and amount of the multiple phases in the system. Dense or lightweight, open or closed porosities, mono- or multi-modal pore size distribution are the typical structural features imparting tailored properties to foams. Furthermore, polymer structuring is used to manipulate properties via orientation and related phenomena (e.g., flow-induced crystallization) [85], and is of utmost effect during the foaming processes.

In fact, in wound healing, the variety of wound types requires a wide range of wound dressings properties and it is not surprising that new products based on polymeric foams are frequently introduced to target different aspects of the healing process. The design opportunity of these special products has paved the way to the introduction of specific healing strategies, and modern wound dressing system based on foamed polymers are much more than just a cover of the wound.

Foams were first introduced in 80’s as substitute for traditional gauze dressing, with the advantages of aforementioned tunability of properties, greater stability (they do not shed particles) and a better control of the wound environment. Foam wound dressing are currently designed in such a way they can be left for several days without causing maceration and with specific healing properties [86]. Key requirements of the dressing are [87]: moisture control, gas permeability, fluid (exudates) transport/absorption, wound protection from microorganisms, necrosis prevention, mechanical protection, movable/removable, wettability, biocompatibility, biodegradability, stiffness, strength, nontoxic, wound pain relief, and cost acceptable. These can be grouped in physical, mechanical, chemical and biological. Table 1 reports the dressing requirement classification.

**Figure 4:**
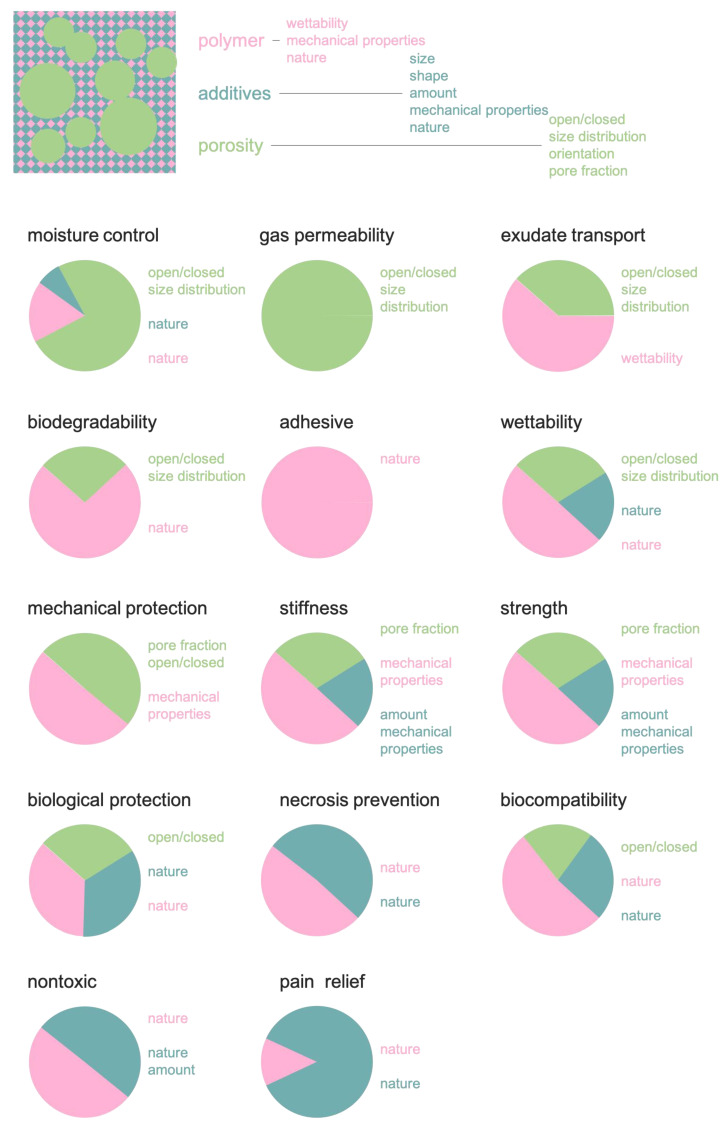
Dependencies of the wound dressing feature upon the foam structure.

The foam structural features described above determine the properties at different extents. For example, the morphology of the pores has a profound effect on permeability or on exudate transport, and to a lesser effect on the mechanical properties, where, instead, foam density has a major role. Figure 4 describes these dependencies.

Based on the type and cause of wound, various products are available in the market, and they are classified as passive, interactive and bioactive products [88]:

- Passive products are non-occlusive used just to cover the wound.
- Interactive dressings, are semi- occlusive or occlusive. Interaction is mainly related to the barrier action against penetration of the bacteria in the wound environment. Semipermeable foam dressings are either hydrophobic or hydrophilic, with adhesive borders for proper positioning.
- Bioactive wound dressings, which are known for their biocompatibility, biodegradability and nontoxic nature, are the latest type and play important, active roles in the healing process, often containing collagen, hyaluronic acid, chitosan, alginate and elastin to this aim. In order to enhance the wound healing process, additives such as growth factors and/or antimicrobials may be incorporated with the base polymer [10].

Several wound dressing systems based on foams are available on the market, reported in Table 2 [62,89].

As shown in Table 2, most of the foams are polyurethane-based, making advantage of the many-decades history of the polyurethane industry and the huge versatility in the chemistry of the polyurethanes. In particular, hydrophilic formulations coupled with open-celled structures, make these products capable of large volumetric exudates retention and inherent absorbent properties. In this way, the wound is kept clear from exudates while maintaining a moist wound bed, which is one of the main features of these products. Other advantages are the tailored mechanical properties. In fact, polyurethanes can be designed to display a rubbery behavior, with a small value of Young’s modulus, thus compliant to conforming to the wound shape and body contour. The open-celled polyurethane foam also prevents trauma, as in mattresses and seats. Thermal insulation properties, which also derive from the porous structure, also help protecting the wound and accelerate healing. Needless to say, unequaled economic advantage is responsible for the large success of polyurethane foamed dressing. Drawbacks, in particular with respect to bulk dressing such as hydrocolloids, are the opacity, which prevents visual monitoring of the wound, and the risk of drying wounds, when little to no exudate is produced, making these products not suitable for dry wounds.

Despite the physiological, mechanical, and economic advantages that polyurethane foams possess over other dressing materials, they as such possess poor healing capabilities and are regarded as a passive wound dressing.

As reported in Table 2, use of additives that can be easily embedded in the formulation may give enhanced properties, both physical (e.g., absorbent, when using polyacrylates or zeolites) and biological (e.g., antimicrobial and antifungal, when using chlorexidine gluconate or polyhexamethylene biguanide or hemostatic, when using chitosan or zeolites) [90]. Furthermore, bioactive additives such as growth factors (e.g., endothelial growth factor), biomolecules (e.g., dextrans) or cells (e.g., keratinocytes, adipose-derived stem cells) have been applied in order to improve their healing capability, particularly for the treatment of complex wounds that cannot be cured with conventional dressings [91]. Silica nanoparticles have proven bioactive and have been tested for polyurethane-based dressings. Silica additives have proven effective in accelerating wound healing by directly or indirectly stimulating the proliferation of fibroblasts. Silica has also been found to directly stimulate the proliferation of human lung fibroblasts without enacting any biosynthetic activity [92]. Furthermore, silica-incorporated dressing materials exhibit enhanced hemostasis abilities and enhanced mechanical properties.

Besides polyurethanes, other polymers of natural and synthetic origin have been the focus of numerous studies. For instance, polyurethane-urea has proven—compared with polyurethane—to have better hydrophilicity and mechanical property due to its stronger hydrogen bonding interaction that results from the two hydrogen donors of the urea moieties. The development of polyurethane-urea as wound dressing foamed matrix is still well beyond that of the polyurethane.

The research in the field of foamed wound dressing is vibrant and new products with novel features and properties continuously emerge. Among others, it is worth citing the use of polyurethane foams with special shape memory features, utilized for embolic applications [90].

Foam dressings can be left on the same place of the wound for several days, without the side effects of maceration. Indeed, this type of foam has the ability to keep the environment safe for wound healing process. Without these good properties, wound pain could deteriorate into a sympathetic nervous system response to the wound pain. Moreover, PU foam is characterized by a microporous upper layer and an internal sponge-like secondary layer. In this manner, it prevents infections and dehydration. Not only is the healing process facilitated, but also it enhances the ability of enzymes during their promotion of epithelialization and control of the biomechanics of the system. The importance of the creation of a microenvironment has been considerably highlighted in the recent years. The bilayered nature of PU wound dressing systems is also able to avoid the formation of bullae.

## 9. Conventional Methods for Foams Production

Sponges or foams can be defined as a dispersion of a gas inside a solid matrix, that can find great applications in the pharmaceutical and biomedical scenario, especially for controlled drug delivery purposes [93]. In particular, alginate and chitosan sponges are characterized by low toxicity, and their ability to regenerate tissues such as cartilage and nerves. Chitosan based composites are currently considered a valuable alternative for wound treatment [94], especially when loaded with antibiotics [95].

Foams can be produced using the thermally induced phase separation, also known as TIPS [96]. According to this technique, the foaming agent is a low boiling organic liquid, that is dissolved into the polymer and determines the phase separation during heating. Then, the nucleation and cell growth occur. The foaming process produced via TIPS is characterized by two main steps: first, a pre-expansion of blowing agent loaded polymer beads is achieved by steam-heating in fluidized flow. Then, pre-expanded beads are deposited to a mold, exposed again to the steam and expanded. During the second expansion step, polymeric pellets sinter together and assume the same shape of the mold in which they had been inserted. Any complex shape can be achieved via this cheap process, or it is possible to cut desired shapes according to the particular applications from blocks [97].

This conventional process whose acronym is TIPS can be also performed using an organic solvent, in which the polymer can be dissolved at high temperatures. As a second step, a quenching step is conducted, obtaining again the phase separation. Of course, the removal of solvent results in one of the challenges of these techniques.

Another possibility to produce foams is by leaching [98,99]. The technique consists in the dissolution of the polymer in a highly volatile solvent, obtaining a solution in a mold loaded with a solid porogen that is usually a water-soluble salt, such as sodium chloride or potassium chloride. Once the solvent has evaporated, the salt can be washed out, leaving a high porous structure inside the polymeric matrix. This technique has a great advantage since it has the ability to obtain a homogeneous distribution of porous size, by tuning the amount of porogen added to the mold. Some disadvantages, such as the presence of contaminant of different kind inside the polymeric foam, or the emission of dangerous substances characterize this method. For these reasons, this technique is not often adopted for the production of foams for the application on wounds.

Moreover, it is necessary to add a consideration among batch and continuous methods of production. Polymeric foams can be made either in batch configuration, or in continuous manner. The batch process is much more employed by the academic research, to study new materials or to assess how to control the properties of the produced foam. This technique has been employed to produce foams of various polymers such as poly(ether imide), poly(ether sulfone), polystyrene, poly(methyl metacrylate) and poly(ethylene vinyl acetate). The parameters affecting the process are soaking time, temperature and blowing agent concentration [100]. However, the idea of making the production on large scale reducing the overall cost is available only on continuous configuration setup.

## 10. Supercritical Methods for Foams Production

The necessity of developing green alternatives to conventional methods made the researchers to think of new substantial variation of the foaming process, substituting the toxic organic solvents with inert and not toxic gases such as argon or carbon dioxide as blowing agents [101]. Therefore, there is another parameter that highly contributes to the properties of the produced foam: the effect of pressure.

As for the conventional method, the high-pressure method is performed in two main steps [102]. In the first one, the polymer is saturated using carbon dioxide at high pressures, and then the expansion occurs. During the saturation, the polymer glass transition temperature decreases, obtaining a sort of polymer plasticization. Indeed, under the glass transition temperature, the polymer is found at the glassy state; while, above this temperature, it will be in the rubbery state. Above the glassy temperature, polymers assume plastic properties; therefore, they can be subjected to deformation without being fractured. Once the polymer is saturated, the pressure is decreased rapidly, carbon dioxide is oversaturated, moving the thermodynamic equilibrium. On this occasion, phase separation and nucleation may occur by increasing the temperature of the system. This will bring the final creation of the polymeric foam. The cell growth will stop at the moment in which the polymeric matrix returns to the glassy state.

The use of CO_2_ results in many advantages [103], such as the possibility to tune solvent power and an enhanced diffusion coefficient. Moreover, the low critical temperature of carbon dioxide (31.1 °C) guarantee a complete separation of carbon dioxide from the polymer, without leaving solvent residue in the polymeric matrix. However, there are two main alternatives for the high-pressure mechanism: saturation of the polymer with CO_2_ at room temperature, followed by gas removal after heating; or, saturation with high pressure and temperature (supercritical conditions), followed by rapid depressurization down to 1 bar. Of course, in these steps the foaming process is dominated by diffusion phenomena.

## 11. Drug Loaded into Foams

Foams can be used to deliver antibiotics to prevent from infections by loading defined amount of drug in the pores matrix system [104]. Moreover, other kind of active molecules can be loaded into foams, in order to enhance enzyme activity and fastening the healing process of the wound. Some examples are reported in the literature and listed in Table 3.

## 12. In Vitro Tests: Definition and Description

Wound healing is a complex process in which several overlapping stages, such as hemostasis and inflammation, migration, proliferation and remodeling, are involved. As a consequence, different cell types (such as growth factors, chemokines, cytokines) are fundamental to achieve a complete and proper healing [113]. Among these cells, keratinocytes, fibroblasts and endothelial cells play a crucial role. Keratinocytes (epithelial cells) must migrate to the wound site to proliferate and differentiate whereas the fibroblasts have to differentiate in myofibroblasts, that are vital regarding tissue formation. Moreover, additional aspects must be considered for achieving a proper treatment. A wound dressing should provide an antibacterial effect and a moist environment as well as having correct properties concerning gas permeability. In this context, this section will explain the most common assays that can be performed to study these wound dressing characteristics, mainly with in vitro tests.

Cell cytotoxicity assay: this test will indicate if the material, or the compound, will reduce the growth of the targeted cells.

MTT (3-(4,5-Dimethylthiazol-2-Yl)-2,5-Diphenyltetrazolium Bromide) is the most used assay and can be used with different type of cells (cancer cell lines, fibroblasts, …). The cells must be firstly cultivated and must be added to a well (in a multi-well plate) where it is already placed the material. After a period of time, the unreacted dye is removed, and the formed crystals (of formazan) are dissolved in DMSO to measure subsequently the absorbance. The cell viability can be calculated with respect to the control value (without the material). A MTT assay can be also used for cell proliferation if the cell percentage is measured for a longer period of time.

Cell migration: the scratch assay is the most common way to determine cell migration [114]. In this case, cells are cultivated, and a scratch is produced on the cells monolayer. Then, the culture medium is removed, and the cells are incubated in presence of the material. The cell migration is evaluated at different times by measuring how the wound is closed.

Immune modulatory activity: macrophages are important in wound healing since they are in charge of killing pathogens (inflammatory response) as well as starting and maintaining tissue regeneration. However, side effects, such as fibrosis, can be produced if the macrophages are incorrectly activated. Moreover, if an external material is introduced in the body, suppressing phagocytosis can be adequate to avoid inflammation and cytokines release. The inflammatory effect can be assessed by NO production via induction of nitric oxide synthase. After cultivating the macrophages cell line, the production of NO is stimulated with lipo-poly(saccharides) as was done in [115,116]. The production of the nitric compound is followed by its transformation in stable nitrite, that can be determined by different analytical techniques.

Furthermore, as was mentioned above, there are additional in vitro experiments that can be useful to determine wound dressing properties regarding its antibacterial activity or drug permeation (if a loaded wound dressing is used).

Antibacterial activity: Two main approaches can be followed to determine the effect of the dressing against bacteria or fungi. One of the approaches is based on an inhibition test. Briefly, the selected bacteria are cultivated on different plates (controls) and then the material is cut (usually a disk-shape) and is placed onto the agar plate where the bacteria are cultivated. If the material has antibacterial activity, bacteria will not grow around the disc [117].

Another approach is based on the cultivation of bacteria in flasks with the subsequent evaluation of the microbial growth with a colorimetric method (control value). At the same time, the bacteria are also cultivated in flasks in which the material is also included. The antibacterial activity can be evaluated by comparing the bacteria growth in presence and in absence of the material [118].

**Figure 5:**
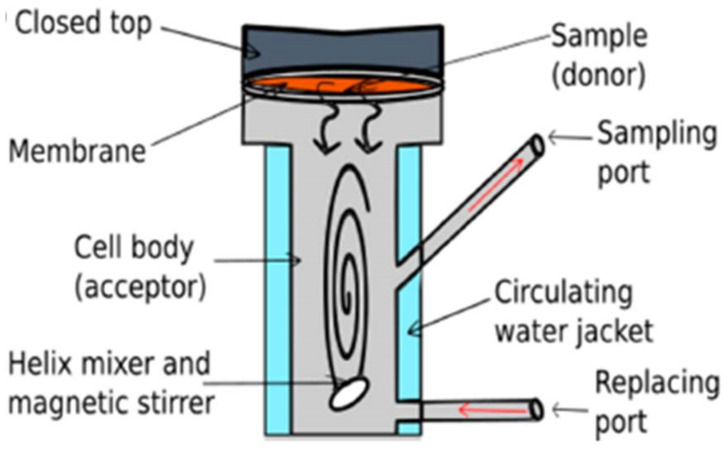
Franz-diffusion cell schematics (adapted with permission from Scientia Pharmaceutica, a MDPI Opena Access Journal [119]).

Drug permeation: the transport of products through the skin is basically a diffusion process in which the layer with the high resistance is the limiting step. This permeation is evaluated with a diffusion cell, mainly Franz type (see Figure 5), that can be static or flow-through. This assay consists in the use of skin (in vitro skin permeation) or a membrane (in vitro release) to separate a donor and receptor chambers. This technique requires a proper selection of the receptor fluid, exposure time and also skin type and integrity. 

More information about this technique can be found in [119,120].

Previous assays are in vitro tests that are useful for an appropriate material characterization. However, it is well-known that this type of tests cannot mimic the different tissues interaction. A more real approximation will be obtained if animal models are used in in vivo experiments. In this aspect, although rodents have been mainly used for these experiments due to their low-cost and availability, it is important to realize that their wound healing mechanism (contract) is different from the human one (re-epithelialization and granulation tissue). According to [121], pig models are more appropriate to predict the results in humans.

In any case, human models will provide more accurate results. Although the main aim of this section is to review the in vitro models, before their application in human models it should be specified that there are different ways to produce wounds in humans. Wilhem et al. reviewed the different wound healing models, such as skin stripping, suction blister, abrasive wound, laser wound, microdermabrasion, dermatome and biopsy, with their respective pros and cons [122].

## 13. In Vitro Tests: Examples and Case Studies

Several articles have reported some of the previous assays to assess the possibility of using different formulations for wound healing applications. As an example, Kukowska et al. [120] used a modification of the tripeptide Gly-His-Lys with fatty acids in in vitro wound healing models. Keratinocyte and human fibroblast cells were used to study cell proliferation, cell migration and cytotoxicity of this compound. MTT assay tests (3-(4,5-Dimethylthiazol-2-Yl)-2,5-Diphenyltetrazolium Bromide) showed that some of these peptides were not cytotoxic towards skin cells providing in addition a proliferative action. Moreover, the migration assay indicated that a high number of the modified peptides were able to reduce the scratch area. Permeation studies using a Franz diffusion cell (Figure 5) also demonstrated that the modified peptides with fatty acids had larger permeability parameters (diffusion and permeability coefficients) than the respective parameters of the compound without modification. These results highlighted the potential of this type of peptides to be loaded in materials for wound dressing systems. On the other hand, although without using foams, the immuno-modulatory activity of another type of material for wound dressings (aerogels with cations) was studied by cultivating a macrophage cell line. This article showed how zinc aerogels were able to reduce NO production and can avoid a future inflammatory response. Although its use is not extended with foams, this NO in vitro assay can provide some valuable information concerning the future response of the macrophages in humans after using a foam for wound healing [122].

Alternatively, the possibility of loading foams, via different techniques, to provide antibacterial activity have been widely explored in the literature. The potential of polyurethane foams with silver hydroxyapatite for wound dressing systems was assessed by Pyun et al. [117]. MTT test with a fibroblast cell line showed no cytotoxicity of the materials. At the same time, the silver release from the foam was determined (ranging from 30 to 120 mg/kg) and the antibacterial activity was studied with an inhibition test against Staphylococcus aureus, Escherichia coli, Pseudomonas aeruginosa and MRSA (Methicillin-Resistant Staphylococcus Aureus). Another example can be the supercritical impregnation of cellulose acetate with thymol, which was released in 21 days in water. A disk inhibition test was performed, indicating a proper activity against bacteria (Escherichia coli and Staphylococcus aureus) and also against fungi (*Candida albicans*) [123].

As a last example, [124] used supercritical CO_2_ to produce in a foam of polycaprolactone that was impregnated in one step with mesoglycan. The article uses keratinocytes, fibroblasts and also endothelial cells to evaluate the effect of the loaded wound dressing. Results showed a positive effect in terms of cytotoxicity and cell migration of the developed material. Moreover, that article proposes the use of a tube formation assay to study endothelial cell organization in a microvascular network, that will give information about the angiogenesis phenomenon [125].

In vivo assays have been also performed with foams. Pyun et al. [126] used male rats Sprague-Dawley, that underwent and an excision on the dorsal area, which was infected with a solution of the bacteria. After that, the polyurethane foam, with silver as antibacterial compound, was put on the wounded area. Then, pictures were taken at different times to evaluate how the wound is reduced, and a subsequent histologic analysis indicated a successful re-epithelialization and collagen formation. More in vivo tests have been done with polyurethane foams. For instance, Lee and Song impregnated that material with povidone-iodine as antibacterial agent and was tested with rats as animal model. They showed with histological analysis and by measuring wound areas how this dressing provided a faster re-epithelialization and angiogenesis than the obtained with other classical dressings [125].

The previous articles show how in vitro and in vivo assays can be used to identify the future effect of the wound dressings after a future application in skin.

However, it is also important to consider that the foams mechanical behavior can play a key role in the healing process. As a matter of fact, fibroblast can synthesize collagen and elastin to provide mechanical strength and elasticity. Moreover, skin is an anisotropic and non-linear viscoelastic material, having an elastic modulus between 0.001 and 57 MPa (depending on the applied strain and strain rates) [126]. Therefore, it would be an advantage to include additional mechanical tests with in vitro and in vivo assays to test the suitability of the developed material if it must mimic skin properties. As an example, Zaman et al. [127], prepared a membrane of gelatin with poly (ethylene-glycol) (PEG) to improve mechanical properties. The membrane was loaded with ciprofloxacin as antibiotic and in vitro antibacterial (inhibition type) and in vivo assays showed that this material was able to heal wounds in rats in 6 days with antibacterial effect. This work showed in addition that the addition of PEG conferred an additional elongation at break while reducing the tensile strength. These results indicated the possibility of modified the mechanical properties by producing composites. In this context, the possibility of using mathematical models to estimate the mechanical properties can be also an advantage to evaluate the future potential of the foam [128].

As a conclusive comment, it is not extended the use of mathematical equations to predict, mainly for loading foams with antibacterial drugs, the bacterial survival curves. Models such as single hit-single target, multiple hit-single target and single hit-multiple target have been defined to predict survival curves of cell lines. Moreover, release models such as Korsmeyer-Peppas [129] have been widely used for investigating the involved mechanisms (such as diffusion or erosion) in the drug release. Coupling these types of equations can be an important advance to be able to predict the antibacterial effect depending on the different drug release mechanisms. An example of the use of these equations can be observed in [130], where an alginate gel with applications as a wound dressing was loaded with polymeric nanoparticles with silver. In that case, a single-hit/single-target model was used with Kornsmeyer-Peppas to predict the survival of different bacteria (*Escherichia coli* and *Bacillus subtilis*) at different times depending on the silver release.

## 14. Conclusions

This work has been focused on the types of dressings used by surgeons and physicians for the treatment of wounds. From the anatomic point of view, wounds had always been considered and treated with great attention, as well as the necessity to create a proper moist environment. Natural and biological steps for wound healing were studied and clarified by medical doctors and researchers in medicine along years, providing a very complex but full knowledge of the phenomena occurring during the restoration of skin in the wounded tissues.

Plants and herbal extracts have always been used for these purposes, but the introduction of antibiotics as drug loaded into dressing systems has represented a huge revolution for the treatment of wounds. In particular, the use of foams has a double advantage: protecting from external attack of viruses and bacteria, and also absorbing huge amounts of exudates. This helps keep the fragile equilibrium in the internal moist environment, while cleaning the wound. High pressure and, in particular, supercritical assisted techniques were recognized to be the greenest methods of production, due to their total absence or reduced presence of solvent residue in the final product, that needs to be applied on the wounded skin, substituting solvents with non-toxic carbon dioxide.

As a take-home message, this work offered a wide panorama of the topic, clarifying that the basic knowledge has been explored completely; now, the applications could be improved more and more, also thanks to the use of foams and other nanoporous materials as medical devices. Moreover, tissue engineering represents one of the most advanced frontiers for wound treatment, especially with the use of stem cells. Stem-cells based therapies could reduce the healing times and decrease patient pain sufferance, while increasing the healing expectancies of chronic wounds, such as pressure ulcers, diabetic foot and removal of necrotic tissues. The addition of computational aided systems could also improve the efficacy in foams production for dressing purposes.

In conclusion, there is still a lack of knowledge of the interaction in cells–foam surface, and that can provide additional and useful information to predict the effect of the dressing on the healing mechanism. Although studies concerning water contact angle and surface charge of the foams have been previously performed, few systematic studies have been done with protein adsorption. These studies, coupled with the previous contact angle and surface properties, can be crucial to know and to predict the effect of the foam regarding for cell proliferation and cell migration, since these phenomena are strongly dependent on material surface properties.

## Figures and Tables

**Table 1:** Dressing requirements and classification.

Physical	Chemical	Mechanical	Biological
Moisture control	Adhesive	Mechanical protection	Protection from microorganism
Gas permeability	Wettability	stiffness	Necrosis prevention
Exudate transport/absorption	---	strength	biocompatibility
Biodegradability	---	----	Nontoxic
---	---	---	Pain relief

**Table 2:** Common foam based wound dressing.

Brand Name	Production Country	Use	Base Polymer	Additive
Flexzan	Dow Hickam Inc., Sugar Land, TX, USA	Chronic wounds	Polyurethane	---
Biopatch	Johnson & Johnson, Malvern, PA, USA	General, with broad-spectrum antimicrobial and antifungal activity	Hydrophilic polyurethane	Chlorhexidine Gluconate
Biatain	Coloplast, Humlebæk, Denmark	Mohs surgery and wounds	Hydrophilic polyurethane	---
Cultinova	Cultinova, München, Germany	Laser resurfacing	Polyurethane	Polyacrylate superabsorbent
Lyofoam	Convatec, Bridgewater, NJ, USA	From moderate to highly exuding wounds	Polyurethane	---
Allevyn	Smith & Nephew, Watford, UK	Chronic or acute exuding wounds	Hydrophilic polyurethane	---
Unilene	Unilene S.A.C., Lima, Peru	Burn and minor injuries	Hydrophilic polyurethane	---
Tielle	3M, St. Paul, MN, USA	Ulcers, post-surgical or traumatic	Silicone and polyurethane foam	---
CuraSpon	CuraMedical B.V., Assendelft, The Nederlands	Hemostasis	Gelatine	---
Kendall	H&R Healthcare Ltd., North Ferriby, UK	General, Bactericidal	Polyurethane	Polyhexamethylene biguanide
Hydrasorb	Hartmann Group, Heidenheim an der Brenz, Germany	General	Polyurethane	---

**Table 3:** Drugs loaded in foams for wound treatment and their applications.

Polymer in the Foam	Drug/Molecule Loaded in the Foam	Field of Application and Effect	Reference
PU	ZnO	Reducing bacterial infection	[105]
Alginate	Azidophyneyl	Drug delivery and wound healing	[106]
Chitosan/alginate	Polyhexamethylene biguanide	Wound dressing	[107]
Alginate	Calcium, Strontium	Drug delivery and tissue engineering	[108]
Alginate	Chitosan, Pluronic F68	biomedical	[109]
Alginate	Hyaluronic acid, chitosan	Tissue engineering	[110]
Alginate	gold nanoparticles, Poly(dimethylsioxane)	biomedical	[111]
Polyurethane/alginate	Jute fiber	Wound dressing	[112]

## Data Availability

No new data were created or analyzed in this study.
